# Functional decline in geriatric rehabilitation ward; is it ascribable to hospital acquired infection? A prospective cohort study

**DOI:** 10.1186/s12877-020-01813-3

**Published:** 2020-10-29

**Authors:** Marie Laurent, Nadia Oubaya, Jean-Philippe David, Cynthia Engels, Florence Canoui-Poitrine, Lola Corsin, Eveline Liuu, Etienne Audureau, Sylvie Bastuji-Garin, Elena Paillaud

**Affiliations:** 1 Univ Paris Est Creteil, INSERM, IMRB, CEpiA Team, F-94010 Creteil, France; 2 AP-HP, Hopital Henri Mondor, Departement de médecine interne et gériatrie, F-94010 Creteil, France; 3 Service de Santé Publique, AP-HP, Hôpital Henri Mondor, F- 94010 Creteil, France; 4 Service de Gériatrie, AP-HP, Hôpital Emile Roux, F- 94450 Limeil Brévannes, France; 5 Univ Paris Est Creteil, Occupational Therapy Institute (IFE), F -94010 Creteil, France; 6 CHU de Poitiers, Service de gériatrie, 2, rue de la Milétrie, F-86021 Poitiers, France; 7 Service de Gériatrie, AP-HP, Hôpital Europeen Georges Pompidou, F-75015 Paris, France

**Keywords:** Comorbidity, Hospitals, Rehabilitation, Elderly, Acquired hospital infection functional decline

## Abstract

**Background:**

In some European countries, including France, older patients with functional decline in acute units are transferred to geriatric rehabilitation units. Some patients may not benefit from their stay in a geriatric rehabilitation unit and paradoxically worsened their functional status. Previous prognostic models of functional decline are based on only baseline parameters. However, some events can occur during rehabilitation and modify the association between baseline parameters and rehabilitation performance such as heart failure episode, falls or hospital-acquired infection (HAI). The incidence of functional decline in these units and factors associated with this decline have not been clearly identified.

**Methods:**

We used a prospective cohort of consecutive patients aged ≥75 years admitted to a geriatric rehabilitation unit in a French university hospital. The main endpoint was functional decline defined by at least an one-point decrease in Activities of Daily Living (ADL) score during the stay. Baseline social and geriatric characteristics were recorded and comorbidities were sought by the Cumulative Illness Rating Scale for Geriatrics (CIRS-G). During follow-up, hospital-acquired infection (HAI) was recorded, as was ADL score at discharge. Multivariate logistic regression and mediation analyses were used to identify factors associated with ADL decrease.

**Results:**

Among the 252 eligible patients, 160 (median age 84 years [interquartile range (IQR) 80–88] had available ADL scores at baseline (median score 7 [IQR 4–10]) and at discharge (median 9 [6–12]). Median CIRS-G score was 11 [8–13], 23 (14%) had a pulmonary HAI; 28 (17.5%) showed functional decline. On multivariable analysis, functional decline was associated with comorbidities (global CIRS-G score, *P* = 0.02, CIRS-G for respiratory disease [CIRS-G-R] ≥2, *P* = 0.02, or psychiatric disease, *P* = 0.02) and albumin level < 35 g/l (*p* = 0.03). Significant associations were found between functional decline and CIRS-G-R (OR 3.07 [95%CI 1.27–7.41], *p* = 0.01), between functional decline and pulmonary HAI (OR 3.12 [1.17–8.32],*p* = 0.02), and between CIRS-G-R and pulmonary HAI (OR 12.9[4.4–37.7], *p* = 0.0001). Theses associations and the reduced effect of CIRS-G-R on functional decline after adjusting for pulmonary HAI (OR 2.26 [0.83–6.16], *p* = 0.11) suggested partial mediation of pulmonary HAI in the relation between CIRS-G-R and functional decline.

**Conclusion:**

Baseline comorbidities were independently associated with functional decline in patients hospitalized in a geriatric rehabilitation unit. Pulmonary HAI may have mediated this association. We need to better identify patients at risk of functional decline before transfer to a rehabilitation unit and to test the implementation of modern and individual programs of rehabilitation outside the hospital for these patients.

## Background

Hospital admissions are important causes of functional decline among older patients [1]. The decline has important effects on quality of life and is associated with increased risk of longer hospital stay, death, nursing home transfer, and rehospitalization [2–4]. Older age, preexisting altered functional status, cognitive impairment, low mobility during the stay and length of stay have been reported to increase the risk of functional decline in acute unit [5–12]. Functional decline can lead to a restriction of participation in meaningful daily activities, which can be dramatic, as studies have shown that the main criteria for frail older adults to express satisfaction with their life is to be able to continue everydays’ occupations and way of living, opposed to using home-care services, first reason given for unsatisfaction [13]. For older adults with cancer for example, studies have shown that autonomy was even more valued than healing or lifespan [14]. Functional Independence Measure (FMI), Barthel’s score and Katz’s Acitiviy of Daily Living score (ADL) have been recognized to have good metrics proprieties and are widely used to assess functional decline. All are multidisciplinary, simple and quick to use. However, some studies suggest that for frail older adults Katz ADL is more appropriate [15]. It is also the tool recommended for best practices in nursing care to older adults [16].

In some European countries, including France, older patients with functional decline in acute units are transferred to geriatric rehabilitation units [1, 17–19] that are inpatient units specialized in the multidisciplinary rehabilitation of older frail patients with chronic diseases and geriatric syndromes [20]. A meta-analysis showed that these units may improve functional status and may limit nursing-home transfer and mortality [21]. One study suggested that some patients may not benefit from their stay in a geriatric rehabilitation unit and paradoxically worsened their functional status [22]. The incidence of functional decline in these geriatric rehabilitation units and factors associated with this decline have not been clearly identified [16]. Previous studies in rehabilitation units focused on specific groups of patients, such as those with hip fracture [22–27] or cognitive impairment [7, 22]. Generalization of results from these studies to the heterogenous population of geriatric rehabilitation units is limited. As compared with younger patients, older patients admitted to a geriatric rehabilitation unit had multiple comorbidities in addition to the primary diagnosis that triggered their admission to the rehabilitation unit [26, 28].

Results concerning comorbidities as predictors of functional outcome for older frail patients are discordant [5, 25–33]. Mechanisms that could tie baseline comorbidities to rehabilitation performance remain unclear [25, 26, 28–32]. Moreover, previous prognostic models are based on only baseline parameters. However, some events can occur during rehabilitation and modify the association between baseline parameters and rehabilitation performance such as heart failure episode, falls or hospital-acquired infection (HAI) which could modify the relationship between the comorbidities and functional decline [34]. Few studies have assessed the link between HAI and functional decline and most of them were performed in acute medical wards. Yet, the incidence of HAI in rehabilitation units is higher than in acute wards [35]. HAIs remain a major cause of morbidity and mortality despite advances in antimicrobial therapy, better supportive care modalities, and the use of a wide range of preventive measures [35].

The first aim of this study was to assess the incidence of functional decline in older patients during a stay in a rehabilitation unit. The secondary aim was to assess association between the functional decline during a stay in a rehabilitation unit and baseline exposition variables such as comorbidities or nutritional status and exposition variables during follow up such as HAI.

## Methods

Our manuscript adhere to the appropriate STROBE guidelines.

### Study design and patients

We used data from a previously described prospective cohort study conducted between July 2006 and November 2008 in a teaching hospital (1300 beds) in the Paris area, France [36]. The cohort comprised 252 consecutive patients aged 75 years or older who were referred to a geriatric rehabilitation unit from acute medical or surgical units during the study period. This rehabilitation unit focuses on minimizing dependency and enabling return to home and participation in community activities of patients with functional impairments that are expected to improve.

In addition to medical care provided by geriatricians and standard nursing care, inpatient rehabilitation typically included both physical therapy (1 h/day) and occupational therapy (1 h/day) on 5 of 7 days per week. Inclusion criteria were medically stable status at admission, need for long-term care and rehabilitation, and absence of terminal disease (severe dementia with Mini-Mental State Examination (MMSE) less than 10 associated with reserved prognosis based on geriatric evaluation), fever, infection, active known malignant process, or known immunological dysfunction. All patients underwent routine assessment by multi-disciplinary staff including physicians, nurses, a physical therapist, an occupational therapist and a social worker. Patients were followed up until death if death occurred during their rehabilitation unit, discharge from the rehabilitation unit if discharge occurred before 3 months after inclusion. If discharge from the rehabilitation unit occurred after 3 months, data of follow up were recorded at 3 months. The study complied with the Declaration of Helsinki and was approved by the Paris XII ethics committee (no. SCR06010), Paris, France. Written informed consent was obtained from each patient before study inclusion.

### Data collection of exposure variables

#### Exposure variables at baseline

Baseline data were collected for each patient, using a standardized form before admission to the geriatric rehabilitation unit: sociodemographic information (age, sex, living conditions) and main acute diagnosis. Comorbidities were evaluated using the Cumulative Illness Rating Scale for Geriatrics (CIRS-G) [37], which scores diseases in 14 organ systems on a 0–4 grading scale of severity (a higher score indicates higher comorbidity). Paper and computerized medical records collected during acute hospitalization were used to define CIRS-G for each patient. The comorbidity index (CIRS-G Index) was calculated as the number of domains with score ≥ 2 and ranged from 0 to 14. The 14 domains were also assessed separately and were considered altered with CIRS-G score ≥ 2. Cognitive function was assessed by the Mini-Mental State Examination (MMSE, score < 24 considered abnormal) [38] and renal function by the Cockroft creatinine clearance (ml/min) [39]. Serum albumin level < 35 g/L was considered low [40].

#### Exposure variables during follow up

During follow-up, 2 of the co-authors (ML and EL) visited each patient once a week and reviewed the medical records with the attending physician and nurses to assess HAI in the rehabilitation unit. HAIs were diagnosed by consensus between 2 investigators (M.L. and E.L.). New onset of HAI was defined as a HAI that was not present at admission and was diagnosed after day 3 of admission in the rehabilitation unit and that met the Centers for Disease Control and Prevention definition of nosocomial infection [41]. Among bacterial infections, only those treated with antibiotics were taken into account; asymptomatic urinary tract infections were not included. When patients experienced more than one HAI, only the first episode was considered in the statistical analysis.

#### Outcome

Functional status was assessed by trained rehabilitation unit staff by the ADL scale, with scores ranging from 0 to 12, 12 indicating no impairment in all 6 activities, (bathing, dressing, toilet use, continence, transfer and feeding). For each activity, the score could be 0 (unable to perform the activity without complete help), 1 (able to perform the activity with little assistance), and 2 (able to perform the activity without any help) [42]. ADL was also assessed at discharge from the rehabilitation unit.

The outcome was functional decline during the rehabilitation unit stay defined by at least a one-point decrease in ADL score using difference between ADL assessed at admission and at discharge from the rehabilitation unit. If discharge from the rehabilitation unit occurred after 3 months, functional decline was recorded at 3 months. We have included all patients admitted to rehabilitation unit independently of their ADL level at baseline (equal or less than 12) to reflect the real life in a rehabilitation unit. Only patients with an ADL level equal to zero at baseline were not included in analysis of factors associated with functional decline because their functional status cannot decrease more.

### Statistical analysis

**Fig. 1:**
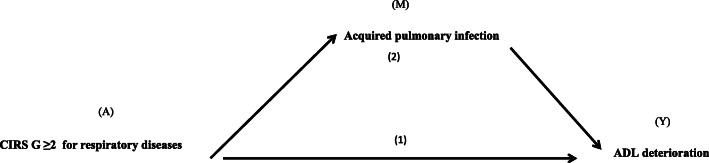
Conceptual framework of the causal structure modelizing mediation. (1) Direct effect of CIRS-G ≥ 2 for respiratory diseases (CIRS-G-R) on activities of daily living (ADL); and (2) indirect effect via acquired pulmonary infection. Legends (A) exposure of interest, (M) mediating factor, (Y) outcome

Categorical variables are described as numbers (%) and were compared by chi-square test or Fisher exact test, as appropriate. Continuous variables are described as median (interquartile range [IQR]) and were compared by the nonparametric Mann-Whitney test. Characteristics of patients with unavailable ADL data at discharge were compared to those with available data. Considering this latter group, we then compared the groups with and without functional decline in terms of baseline characteristics. In univariate analysis, associations were assessed using logistic regression model and crude odds ratios (ORs) were estimated with their 95% confidence intervals (95% CIs). Bivariate analyses were performed to identify the potential confounding factors. The assumption of loglinearity for continuous variables was checked using likelihood ratio tests comparing models with the variable handled in continuous versus in categorical way. We used the same methodology to test interactions. We looked for relevant interactions based on the literature: we tested interaction between main acute diagnosis and baseline ADL, between Pulmonary HAI and CIRS-G for respiratory disease, between Acquired Pulmonary Infection and CIRS-G Index and between MMSE. Variables associated with functional decline on univariate analysis at *P* < 0.15 were then entered into a multivariate logistic regression model. The model was built using manual stepwise approach, (manual backward and forward approaches). To avoid introducing strongly correlated variables into multivariate models, we assessed correlations by using Cramer’s V for categorical variables and the nonparametric Spearman’s rank correlation coefficient (Rho) for quantitative variables using a correlation matrix. All models including albumin level were systematically adjusted for C-reactive protein (CRP) level, as appropriate [40]. Calibration of the final model was assessed using Hosmer-Lemeshow test. Finally, and in accordance with our hypothesis, we examined whether HAI occurrence potentially mediated the relation between comorbidities and functional decline, as illustrated in the conceptual framework shown in Fig. 1. According to Baron and Kenny [43], evidence for a partial mediating effect was assessed by the statistical significance [43–46] of the following associations:

1. between comorbidities as the independent exposure of the interest (A) and functional decline as the outcome (Y),
2. between comorbidities and HAI as the mediating factor (M),
3. and between HAI and functional decline and by a reduced effect of comorbidities on functional decline after adjusting for HAI.

### Sensitivity analyses

To test the robustness of our results, we performed three sensitivity analyses on the final models. Using the hypothesis of maximal bias, we first considered that all patients with missing discharged ADL data had functional decline, and second that these patients had no functional decline. Finally, we used a multiple imputation approach with the multiple-multivariate imputation-by-chained-equations procedure with the missing-at-random assumption. We used all baseline covariates and outcomes together to impute missing data values and independently analyzed 20 copies of the data.

All tests were two-tailed. *P* ≤ 0.05 was considered statistically significant. Data were analyzed by using STATA v11.0 (StataCorp, College Station, TX, USA).

## Results

**Fig. 2:**
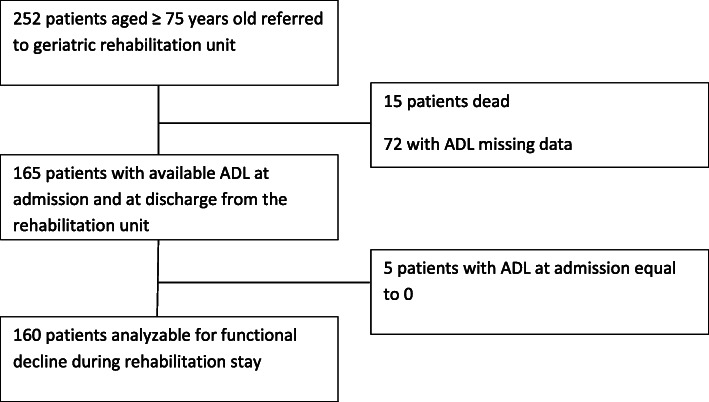
Flow chart of the study. Functional decline was defined by at least a one-point decrease in ADL score using difference between ADL assessed at admission and at discharge from the rehabilitation unit

The 15 patients who died during their stay in the rehabilitation unit were not included in the present study. Among the remaining 237 patients, 72 (30.3%) had missing ADL data at discharge (Fig. 2). These patients were older, had lower admission ADL values, a higher number of comorbidities and a higher rate of HAI during rehabilitation stay than the 165 patients with available data (all *p* < 0.05) (supplemental data – Appendix 1).

Among the 165 patients with available ADL data at discharge, 5 patients had an ADL score equal to 0 at baseline, they were excluded from the analysis of factors associated with functional decline due to the inability to detect ADL deterioration in these patients. In patients analyzed for factors associated with functional decline, at baseline 17 patients had an ADL score equal to 12 and 143 patients had an ADL score of less than 12. Median ADL at discharge from the rehabilitation unit was significantly lower for patients with than without functional decline (4.5 [1–9] vs 10 [6–12] *p* < 0.0001). In study population, incidence of functional decline between admission in rehabilitation unit and discharge from rehabilitation unit was 17.5%. Forty-Eight (30%) had a HAI (Table 1), 42.9% in functional decline group and 27.3% in group without functional decline (*p* = 0.11), 120 (75%) patients had CIRS-G score ≥ 2 for psychiatric diseases (89.3% in functional decline group and 72% in group without functional decline, *p* = 0.06) and 93/120 patients with CIRS-G score ≥ 2 for psychiatric diseases had MMS < 25. Median length of unit stay was 29 days [15–63], with no difference between the groups with and without functional decline (median 35.5 days [14.5–79] vs 29 days [15–60], *P* = 0.80). Twenty-eight patients had stayed longer than 3 months, maximum length of stay was 221 days. All other variables that were compared between the groups with and without functional decline are presented in Table 1. On univariate analysis, global CIRS-G and CIRS-G Index were significantly higher for patients with than without functional decline. Among the CIRS-G categories, respiratory and psychiatric diseases were significantly more prevalent in patients with functional decline. These patients had also lower MMSE values and low albumin level. The occurrence of pulmonary HAI was significantly associated with functional decline (OR 3.12 [1.17–8.32], *p* = 0.02) but also with CIRS-G ≥ 2 for respiratory diseases (OR 14.98 [4–83-46.4],*p* < 0.0001). There was no interaction between pulmonary HAI and CIRS-G Index (p LR test = 0.83), between pulmonary HAI and CIRS-G for respiratory disease (p LR test = 0.90), between main acute diagnosis and baseline ADL (p LR test = 0.31), and between pulmonary HAI and MMSE (p LR test = 0.71). Because of co-linearity between overall (CIRS-G Index) and specific measures of severe comorbidities (CIRS-G ≥ 2 for respiratory rho 0.34, *p* < 0.0001 or psychiatric diseases rho 0.22, *p* < 0.005), we created two separate models, with the CIRS-G Index or with specific CIRS-G domains. MMSE, strongly associated with CIRS-G ≥ 2 for psychiatric diseases (rho 0.41, *p* < 0.0001), was not introduced in the model with specific domains.

**Table 1:** Characteristics of older patients with or without functional decline during a rehabilitation unit stay and associated factors

		Functional decline^a^ during rehabilitation unit stay			
	Study population *n* = 160 (%)	Yes *n* = 28 (%)	No *n* = 132 (%)	Crude OR [95% CI] †	*P* value ‡
**Baseline characteristics**					
Age, years, median [Q1-Q3]	84 [80–88]	83 [81–87.5]	84 [80–88]		0.78
Male sex	44 (27.5)	9 (32.1)	35 (26.5)		0.55
Living alone	110 (68.8)	19 (67.9)	91 (68.9)		0.91
Place of residence					
Home or assisted-living facility	155 (96.9)	27 (96.4)	128 (97.0)		0.88
Nursing home	5 (3.1)	1 (3.6)	4 (3.0)		
Main acute diagnosis					0.48
Cardiovascular diseases	36 (22.5)	6 (21.4)	30 (22.7)		
Cerebrovascular diseases	50 (31.2)	12 (42.9)	38 (28.8)		
Orthopedic diseases (including fracture)	35 (21.9)	4 (14.3)	31 (23.5)		
Other diagnosis^b^	39 (24.4)	6 (21.4)	33 (25.0)		
ADL at admission in rehabilitation unit, median [Q1-Q3]	7 [4–10]	5 [3–10]	7.5 [4–10]	0.91 [0.81–1.03]	0.14
**Comorbidities**					
Global CIRS-G, median [Q1-Q3], OR/_1-point increase_ (*n* = 164)	11 [8–13]	12.5 [10–15.5]	10 [8–12]	1.15 [1.03–1.28]	0.01
CIRS-G Index, median [Q1-Q3], OR/_1-point increase_	4.0 [3.5–5.5]	6 [4–6]	4 [3–5]	1.46 [1.14–1.86]	0.003
Number of patients with CIRS-G score ≥ 2 in each category, n (%)					
Cardiovascular/respiratory system					
Heart disease	101 (63.1)	20 (71.4)	81 (61.4)		0.32
Hypertension	116 (72.5)	19 (66.9)	97 (73.5)		0.55
Vascular/hematological diseases	44 (27.5)	9 (32.1)	35 (26.5)		0.55
Respiratory diseases	34 (21.3)	11 (39.3)	23 (17.4)	3.07 [1.27–7.41]	0.01
Eye, ear, nose and larynx diseases	36 (22.5)	6 (21.4)	30 (22.7)		0.88
Gastrointestinal system					
Upper gastrointestinal diseases	12 (7.5)	3 (10.7)	9 (6.8)		0.48
Lower gastrointestinal diseases	13 (8.1)	3 (10.7)	10 (7.6)		0.58
Hepatic diseases	1 (0.6)	0 (0)	1 (0.8)		–
Genitourinary system					
Renal diseases	56 (35)	14 (50)	42 (31.8)	2.14 [0.94–4.90]	0.07
Other urogenital diseases	33 (20.6)	8 (28.6)	25 (18.9)	1.71 [0.68–4.33]	0.26
Musculoskeletal/intergumentary system					
Muscle, bone, and skin diseases	80 (50.0)	16 (57.1)	64 (48.5)		0.41
Neuropsychiatric system					
Neurological diseases	42 (26.3)	10 (35.7)	32 (24.2)		0.21
Psychiatric diseases	120 (75.0)	25 (89.3)	95 (72.0)	3.25 [0.92–11.40]	0.06
General system					
Endocrine and metabolic diseases	44 (27.5)	10 (35.7)	34 (25.8)		0.29
MMSE, median [Q1-Q3], OR/_1-point decrease_	22 [17–26]	18 [15–25]	23 [18–27]	1.10 [1.02–1.18]	0.02
MMSE < 24	87 (56.5)	19 (70.4)	68 (53.5)	2.06 [0.84–5.05]	0.11
Albumin level < 35 g/L	86 (53.5)	21 (75)	65 (49.2)	3.09 [1.23–7.77]	0.02
CRP level, mg/L, median [Q1-Q3]	6 [2.5–13]	8 [2.5–17.5]	5 [2.5–12.5]		0.59
Creatinine clearance Cockroft, ml/min, median [Q1-Q3], OR/_1-point decrease_ (n = 164)	41.5 [32.6–54.1]	50.2 [30.5–55.8]	40.4 [32.7–53.3]		0.57
**Hospital-acquired infection (HAI)**					
Acquired infection during rehabilitation period ^c^	48 (30.0)	12 (42.9)	36 (27.3)	2.00 [0.86–4.64]	0.11
Pulmonary HAI	23 (14.4)	8 (28.6)	15 (11.4)	3.12 [1.17–8.32]	0.02
Acquired urinary infection	23 (14.4)	5 (17.9)	18 (13.6)		0.56
Other acquired infections	5 (3.1)	0 (0)	5 (3.8)		–

*Abbreviation*: *OR* Odds ratio, *CI* Confidence interval, *ADL* Activities of daily living, *CIRS-G* Cumulative Illness Rating Scale for Geriatrics, *CRP* C--reactive protein, *MMSE* Mini-Mental State Examination

The CIRS-G consists of 14 domains related to different body systems. Scoring on the different domains is weighted by the severity of the comorbid condition. Severity scores range from 0 (none) to 4 (extremely severe). The global score is the sum of each of the 14 domain scores. The CIRS-G index was calculated as the number of categories with score ≥ 2

(n=) indicates the number of patients with available data

‡*P* value by logistic regression (Wald test)

^a^Functional decline was defined by at least a one-point decrease in ADL score during the rehabilitation unit stay

^b^Including respiratory, gastrointestinal, and osteoarticular disease other than fracture

^c^Some patients had two or more acquired infections, so the sum of the patients in the three acquired infection groups is > 48

Table 2 shows factors independently associated with functional decline during the rehabilitation unit stay. On multivariate analysis, functional decline was significantly associated with the CIRS-G Index and low MMSE values, with a non-significant association for low albumin level (Table 2, model 1). Adjustment for CRP was forced in models including albumin level, as CRP may be a confounder in the association between albumin level and functional decline. *P*-value of Hosmer-Lemeshow test for model 1 was 0.49 indicating good calibration. Two CIRS-G–specific domains, namely respiratory and psychiatric diseases, and low albumin level were independently associated with functional decline (Table 2, model 2). Systematic adjustment for admission ADL value did not change the results (data not shown). After adjustment for CIRS-G index or specific respiratory or psychiatric CIRS-G, pulmonary HAI was not significantly associated with functional decline. Therefore pulmonary HAI was not kept in final models. ADL score at baseline, CIRS-G ≥ 2 for renal or other urogenital diseases were no longer significantly associated with functional decline after adjustment for CIRS-G index or CIRS-G for respiratory or psychiatric diseases and were not kept in final models. *P*-value of Hosmer-Lemeshow test for model 2 was 0.39 indicating good calibration.

**Table 2:** Factors independently associated with deteriorated activities of daily living (ADL) during the rehabilitation unit stay

	Model 1 Adjusted OR [95%CI]	*P* value	Model 2 Adjusted OR [95%CI]	*P* value
CIRS-G Index	1.38 [1.06–1.81]	0.02	_	_
CIRS-G score ≥ 2 for respiratory diseases	_	_	3.23 [1.21–8.59]	0.02
CIRS-G score ≥ 2 for psychiatric diseases	_	_	4.89 [1.27–18.72]	0.02
Albumin level < 35 g/l	2.65 [0.98–7.10]	0.05	2.98 [1.12–7.92]	0.03
MMSE_1-point decrease_	1.09 [1.00–1.18]	0.03	_	_
CRP	0.99 [0.96–1.02]	0.55	0.99 [0.97–1.02]	0.56

Adjusted ORs were estimated by logistic regression adjusted for CIRS-G index, Albumin level and MMSE for model 1 and adjusted for CIRS-G score ≥ 2 for respiratory diseases, CIRS-G score ≥ 2 for psychiatric diseases and Albumin level for model 2. (P value: Wald test)

MMSE Mini-Mental State Examination; CIRS-G Cumulative Illness Rating Scale for Geriatrics, CIRS-G Index calculated as the number of categories with score ≥ 2. Model 1 considers factors associated with ADL deterioration and is adjusted by CIRS-G index, albumin level < 35 g/l, and MMSE. Model 2 considers factors associated with ADL deterioration and is adjusted for CIRS-G score ≥ 2 for respiratory and psychiatric diseases and albumin level < 35 g/l.

Significant or trend associations were observed 1/ between CIRS-G ≥ 2 for respiratory diseases and functional decline (crude OR 3.07 [1.27–7.41] 2/ between CIRS-G ≥ 2 for respiratory diseases and pulmonary HAI (OR 14.98 [4–83-46.4] *p* < 0.0001), and 3) between pulmonary HAI and functional decline (OR 3.12 [1.17–8.32]). The reduced effect of CIRS-G ≥ 2 for respiratory diseases on functional decline observed after adjusting for pulmonary HAI (OR 2.26 [0.83–6.16] *p* = 0.11) (Table 2) suggested partial potential mediation of acquired pulmonary HAI in the relation between CIRSG ≥2 for respiratory diseases and functional decline.

### Sensitivity analyses

The three sensivity analyses produced similar results (supplemental data – Appendix 2).

## Discussion

Among patients 75 years and older referred to a geriatric rehabilitation unit from acute medical or surgical units, 17.5% had functional decline during their hospitalization in the rehabilitation unit. Factors independently associated with functional decline were comorbidities assessed by the CIRS-G index and specifically the CIRS-G ≥ 2 for respiratory or psychiatric diseases. This highlights the need of having a holistic and multiprofessional approach, centered on the person, to include all the dimension of the person from the assessment till the discharge, rather than a pure disciplinary-approach [34]. Our results also suggest that pulmonary HAI in patients hospitalized in rehabilitation units may mediate the relation between CIRS-G ≥ 2 for respiratory diseases and functional decline.

To our knowledge, no previous study has estimated the incidence of acquired functional decline, assessed by ADL, in older patients during a rehabilitation unit stay. In previous studies, functional improvement was assessed with different tools such as the Functional Independence Measure [22, 23, 25, 26, 29, 33] or the Barthel Index [27]. Comparing studies is difficult because of the heterogeneity of these tools. In keeping with two previous studies [33, 47], advanced age was not associated with functional decline.

In our study, comorbidities assessed by the CIRS-G, particularly severe psychiatric and respiratory diseases, were significant predictors of functional decline during the rehabilitation stay. One meta-analysis [26] showed that results concerning the association between functional decline and comorbidities are discordant. Tools used to assess comorbidities are heterogeneous. The main tools used are the Charlson comorbidity index, the comorbidity Index of Liu, the Comorbidity Severity Index or the CIRS-G. Only studies assessing comorbidities with indexes taking into account the severity of diseases such as the CIRS-G and not simple counts of comorbidities found an association between comorbidities and functional status [25, 28–31].

The proportion of HAI in our study was higher than one would expect in some geriatric rehabilitation. We can assume that in our study, physicians who collected data of HAI were more aware of diagnosis of HAI than other doctors because HAI was the main outcome of the cohort study [36]. One hypothesis could be the underreported of HAI in others studies as mentioned in one review [48]. The high proportion of patients with cerebrovascular diseases in our study could also explain this difference because patients with cerebrovascular diseases are more likely to have HAI especially pulmonary HAI [49]. Patients whose acute event was cerebrovascular disease seemed to experience more frequently functional decline than those whose acute event was orthopedic disease; this may reflect that cerebrovascular disease affects risk of functional decline and the potential link between HAI and functional decline.

Our findings are consistent with previous studies showing that cognitive impairment and depressive symptomatology predicted poor rehabilitation [7, 50, 51]. Similarly, in one cohort of 459 older patients hospitalized in a general medical service, risk of 1-month functional decline was two- to three-fold higher for patients with depression, delirium or with the overlap syndrome of depression and delirium than patients with neither depression nor delirium [52]. In our population, specific psychiatric CIRS -G concerned a major part of patients with cognitive dysfunction. The MMSE scores of the study population were lower than one would expect in a rehabilitation unit. Measure of cognitive function at rehabilitation admission could underestimate the true cognitive potential of patients.

The CIRS-G ≥ 2 for respiratory diseases was associated with functional decline and with pulmonary HAI. Reduced ability to participate in physiotherapy cessions due to respiratory diseases may lead to peripheral muscle dysfunction and therefore functional decline [53]. An association between chronic respiratory diseases and pulmonary HAI was previously described in one Spanish study of a subacute care unit with frail older patients [32]. Inflammatory cytokine levels (tumor necrosis factor α) are increased with pulmonary infection and were previously found to be associated with functional decline [54].

We found an association between low serum albumin level upon admission and functional decline persisting after adjustment for an inflammation marker (CRP level). Protein-calorie malnutrition, frequent in older people, leads to muscular loss and may explain this association [55].

Our study has certain strengths. Comorbidities were measured for all participants at baseline and were assessed by using a formal, validated and standard scale of comorbidity taking into account the severity of chronic diseases. This is the first study to analyze the association between functional decline and specific domains of the CIRS-G. This may be the first approach to explain the relation between functional decline and comorbidities, taking into account HAI.

However, the study contains several limitations. First, the single-center design of our study may have led to recruitment bias, thereby limiting external validity. Detailed operational characteristics, such as the intensity and frequency of physical therapy or other functional measure such as handgrip test, were not available for each patient [21]. Factors other than HAI occurring during the hospital stay could be involved in functional decline and we cannot exclude residual confounding factors in this cohort study. We did not analyze the impact of functional decline on future hospital re-admission, quality of life, and social costs. Finally, from the initial cohort of 252 consecutive patients, only data for 160 were analyzed, which may have led to selection bias. However similar results obtained by sensitivity analyses support the robustness of our findings.

### Implications

The goals of the rehabilitation unit for older patients are to achieve significant functional improvement mainly in mobility, thereby enabling these patients to return home in a relatively short time. However, we found that these tasks can be difficult to achieve in older patients with severe respiratory or cognitive impairment. We need to better identify patients at risk of functional decline before transfer to a rehabilitation unit. Having a HAI could influence relation between comorbidities and functional decline. We need to improve hygiene measures and the prevention of HAI in this population. It would be interesting to test in France the implementation of modern and individual programs of rehabilitation outside the hospital, such as those from Norway, Sweden and Spain [56–58] in particular for patients at-risk of HAI and hospital functional decline. Home-based occupational therapy, notably because occupational therapy has a person-centred approach focusing on the patient’s own valued daily-activities, could improve functioning in frail older adults [59].

## Conclusion

Among patients 75 years and older referred to a geriatric rehabilitation unit from acute medical or surgical units, incidence of functional decline during their hospitalization in rehabilitation unit was almost 20%. Factors associated with functional decline were severe respiratory or cognitive impairment. Pulmonary Hospital-acquired infection in patients hospitalized in rehabilitation units may mediate the relation between respiratory diseases comorbidities and functional decline.

## Supplementary information

**Additional file 1 **: **Appendix 1**. Tableau A1. Characteristics of older patients with or without Activites of Daily Living (ADL) missing. **Appendix 2**. Tableau A2. Factors independently associated with ADL deterioration during rehabilitation unit stay, sensitivity analyses.

## Data Availability

The datasets used and/or analysed during the current study are available from the corresponding author on reasonable request. All data generated or analysed during this study are included in this published article [and its supplementary information files].

## Abbreviations

ADL: Activities of Daily Living
CIRS-G: Cumulative Illness Rating Scale for Geriatrics
CRP: C-reactive protein
EL: Evelyne Liuu
HAI: Hospital-Acquired Infection
ML: Marie Laurent
MMSE: Mini-Mental State Examination
IQR: InterQuartile Range
ORs: Odds Ratios
