# Supplementary material for: Functional decline in geriatric rehabilitation ward; is it ascribable to hospital acquired infection? A prospective cohort study
Source: BMC Geriatr. 2020 Oct 29;20:433. doi: 10.1186/s12877-020-01813-3 (PMC7597031; doi:10.1186/s12877-020-01813-3)
Supplement: Supplementary file 1 — Additional file 1 : Appendix 1. Tableau A1. Characteristics of older patients with or without Activites of Daily Living (ADL) missing. Appendix 2. Tableau A2. Factors independently associated with ADL deterioration during rehabilitation unit stay, sensitivity analyses. [file 12877_2020_1813_MOESM1_ESM.docx]

Supplemental data

Appendix 1. Tableau A1. Characteristics of older patients with or without Activites of Daily Living (ADL) missing.

|  | Study population  n=160 |  | | | Population  with ADL missing  n=71 | | P value | |
| --- | --- | --- | --- | --- | --- | --- | --- | --- |
| **Baseline characteristics** |  |  | | |  | |  | |
| Age, years, median [Q1-Q3] | 84 [80-88] |  | | | 86 [83-91] | | **0.008** | |
| Male gender | 44 (27.5) |  | | 21 (29.6) | | 0.74 | |  |
| Living alone | 110 (68.8) |  | | 50 (70.4) | | 0.79 | |  |
| **Place of residence:** |  |  | |  | | 0.44 | |  |
| Home or assisted-living facility | 155 (96.9) |  | | 70 (98.6) | |  | |  |
| Nursing home | 5 (3.1) |  | | 1 (1.4) | |  | |  |
| **Main acute diagnosis** |  |  | |  | | 0.85 | |  |
| Cardiovascular diseases | 36 (22.5) |  | | 12 (17.6) | |  | |  |
| Cerebrovascular diseases | 50 (31.2) |  | | 22 (32.3) | |  | |  |
| Orthopedic diseases (including fracture) | 35 (21.9) |  | | 15 (22.1) | |  | |  |
| Other diagnosis† | 39 (24.4) |  | | 22 (32.3) | |  | |  |
| ADL at admission in rehabilitation unit, median [Q1-Q3] | 7 [4-10]- |  | | 7 [4-10] | | 0.57 | |  |
| **Comorbidities** |  |  |  | | |  |  |  |
| Global CIRS G_1 point increase_, median [Q1-Q3], (n=164) | 11 [8-13] |  | | 13[10-15] | | | **0.001** | |
| Comorbidity index, median [Q1-Q3] | 4.0 [3.5-5.5] |  | | 5[4-6] | | | **0.001** | |
| Cardiovascular/respiratory system |  |  | |  | | |  | |
| 1. Heart disease | 101 (63.1) |  | | 52(73.2) | | | 0.13 | |
| 1. Hypertension | 116 (72.5) |  | | 54(76.1) | | | 0.57 | |
| 1. Vascular/hematological diseases | 44 (27.5) |  | | 24(33.8) | | | 0.33 | |
| 1. Respiratory diseases | 34 (21.3) |  | | 18(25.3) | | | 0.49 | |
| 1. Eye, ear, nose and larynx diseases | 36 (22.5) |  | | 23(32.4) | | | 0.11 | |
| Gastrointestinal system |  |  | |  | | |  | |
| 1. Upper gastrointestinal disease | 12 (7.5) |  | | 3(4.2) | | | 0.35 | |
| 1. Lower gastrointestinal disease | 13 (8.1) |  | | 11(15.5) | | | **0.09** | |
| 1. Hepatic diseases | 1 (0.6) |  | | 1(1.4) | | | 0.55 | |
| Genitourinary system |  |  | |  | | |  | |
| 1. Renal diseases | 56 (35) |  | | 33(46.5) | | | 0.10 | |
| 1. Other urogenital diseases | 33 (20.6) |  | | 18(25.3) | | | 0.42 | |
| Musculoskeletal/integumentary system |  |  | |  | | |  | |
| 1. Muscle, bone, and skin diseases | 80 (50.0) |  | | 53(74.6) | | | **<0.0001** | |
| Neuropsychiatric system |  |  | |  | | |  | |
| 1. Neurological diseases | 42 (26.3) |  | | 16(22.5) | | | 0.55 | |
| 1. Psychiatric diseases | 120 (75.0) |  | | 60(84.5) | | | 0.10 | |
| General system |  |  | |  | | |  | |
| 1. Endocrine and metabolic diseases | 44 (27.5) |  | | 21(29.6) | | | 0.75 | |
| MMSE_1 point decrease_, median [Q1-Q3] | 22 [17-26] |  | | 21[16.5-24] | | | 0.14 | |
| Albumin level <30 g/L | 31 (18.8) |  | | 16(22.5) | | | 0.43 | |
| Albumin level <35 g/L | 86 (53.7) |  | | 47(66.2) | | | **0.07** | |
| CRP , mg/L, median [Q1-Q3] | 6 [2.5-13] |  | | 7[2.5-26] | | | 0.20 | |
| Creatinine clearance Cockroft, ml/min _1 point decrease_ ,  median [Q1-Q3] (n=164) | 41.5[32.6-54.1] |  | | 39.7[30.7-50.6] | | | 0.17 | |
| **ADL at discharge from rehabilitation unit** | 9 [6-12]- |  | |  | | |  | |
| **Hospital-acquired infection** |  |  | |  | | |  | |
| Infection during rehabilitation period ‡ | 48 (30.0) |  | | 35(49.3) | | | **0.005** | |
| Pulmonar infection | 23 (14.4) |  | | 21 (29.5) | | | **0.007** | |
| Urinary infection | 23 (14.4) |  | | 13(18.3) | | | 0.44 | |

Functional decline in rehabilitation was defined by decreasing ADL during rehabilitation unit stay.

Crude OR are reported for variables associated with deteriorated ADL at p<0.15. For patients with missing data, number of patients with available variable was defined (n=).

* by chi-square test, Fisher exact test, or Kruskal Wallis test as appropriate.

†Including respiratory disease, gastrointestinal disease, osteoarticular disease other than fracture.

‡Some patients had two or more acquired infections, so the sum of patients in the three groups is greater than 51.

ADL Activities of Daily Living on 12 points; CIRS-G Cumulative Illness Rating Scale for Geriatrics. Severe CIRS was defined by CIRS grade 3-4

CRP, C-reactive protein; MMSE, Mini-Mental State Examination, MD: Missing Data

The CIRS –G consists of 14 domains related to different body systems. Scoring on the different domains is weighted by the severity of the comorbid condition. Severity scores range from 0 (none) to 4 (extremely severe). The global score is the sum of each of the 14 individual domain scores. The CIRS-G Index was calculated as the number of categories with score ≥ 2.

Appendix 2. Tableau A2. Factors independently associated with ADL deterioration during rehabilitation unit stay, sensitivity analyses.

|  | Model 1  Adjusted OR [95% CI] | *P* value | Model 2  Adjusted OR [95% CI] | *P* value |
| --- | --- | --- | --- | --- |
| **Sensitivity analysis no. 1** |  |  |  |  |
| CIRS-G Index | 1.36 [1.12-1.63] | 0.001 | _ | _ |
| CIRS G ≥2 for respiratory diseases | _ | _ | 1.76 [0.90-3.42] | 0.10 |
| CIRS G ≥2 for psychiatric diseases | _ | _ | 2.56 [1.25-5.22] | 0.01 |
| Albumin level < 35 g/l | 1.73 [0.96-3.15] | 0.07 | 2.09 [1.18-3.72] | 0.01 |
| MMSE_1 point decrease_ | 1.05 [0.99-1.10] | 0.09 | _ | _ |
| **Sensitivity analysis no. 2** |  |  |  |  |
| CIRS-G Index | 1.38 [1.06-1.81] | 0.017 |  |  |
| CIRS G ≥2 for respiratory diseases |  |  | 3.23 [1.21-8.59] | 0.02 |
| CIRS G ≥2 for psychiatric diseases |  |  | 4.88 [1.27-18.72] | 0.02 |
| Albumin level < 35 g/l | 2.64 [0.98-7.10] | 0.05 | 2.98 [1.12-7.92] | 0.03 |
| MMSE_1 point decrease_ | 1.09 [1.00-1.18] | 0.03 |  |  |
| **Sensitivity analysis no. 3** |  |  |  |  |
| CIRS-G Index | 1.38 [1.10-1.74] | 0.006 |  |  |
| CIRS G ≥2 for respiratory diseases |  |  | 2.76 [1.05-7.21 ] | 0.04 |
| CIRS G ≥2 for psychiatric diseases |  |  | 4.14 [1.34-12.8] | 0.01 |
| Albumin level < 35 g/l | 1.79 [0.73-4.42] | 0.20 | 2.11 [0.90-4.98] | 0.08 |
| MMSE_1 point decrease_ | 1.06 [0.98-1.15] | 0.09 |  |  |
|  |  |  |  |  |

Sensitivity analysis 1: considering that patients with missing discharged ADL data had a functional decline

Sensitivity analysis 2: considering that patients with missing discharged ADL data had no functional decline

Sensitivity analysis 3: analysis with multiple multivariate imputations for missing discharged ADL data

Adjusted ORs were estimated by logistic regression adjusted for all variables in the table, with routine adjustment for CRP level (*P* value: Wald test).

MMSE, Mini- Mental State Examination; CIRS-G, Cumulative Illness Rating Scale for Geriatrics, CIRS-G Index calculated as the number of categories with score ≥ 2.
